# Soft tissue facial changes among adult females during alignment stage of orthodontic treatment: a 3D geometric morphometric study

**DOI:** 10.1186/s12903-021-01425-2

**Published:** 2021-02-09

**Authors:** Si Yu Hou, Wenwen Zhou, Hongwei Dai, Hai Ming Wong, Yi Feng Wen, Jianping Zhou

**Affiliations:** 1 Stomatological Hospital of Chongqing Medical University China, No. 426 Songshi North Road, Chongqing, China; 2 Chongqing Key Laboratory of Oral Diseases and Biomedical Sciences, Chongqing, China; 3 Chongqing Municipal Key Laboratory of Oral Biomedical Engineering of Higher Education, Chongqing, China; 4 Paediatric Dentistry, Faculty of Dentistry, The University of Hong Kong, China, 34 Hospital Road, Hong Kong, Hong Kong; 5 Key Laboratory of Shaanxi Province for Craniofacial Precision Medicine Research, College of Stomatology, Xi’an Jiaotong University., No. 98 Xiwu Road, Xi’an, Shaanxi Province China

**Keywords:** Soft tissue, Face, Orthodontic treatment, Geometric morphometrics, Quasi-landmarks

## Abstract

**Background:**

To investigate changes in facial morphology during the first six months of orthodontic treatment among adult females receiving orthodontic treatment.

**Methods:**

43 adult females receiving orthodontic treatment were randomly recruited. 3D facial images were taken at baseline (T0), three months (T1), and six months (T2) after treatment initiation. Spatially dense facial landmarks were digitized to allow for sufficient details in characterization of facial features. 3D geometric morphometrics and multivariate statistics were used to investigate changes in mean and variance of facial shape and facial form associated with treatment.

**Results:**

We observed statistically significant changes in facial shape across the three treatment stages (*p* = 0.0022). Pairwise comparisons suggested significant changes from T0 to T1 (*p* = 0.0045) and from T0 to T2 (*p* = 0.0072). Heatmap visualization indicated that the buccal and temporal region were invaginated while the labial region became protruded with treatment. The magnitude of shape change was 0.009, 0.004, and 0.010 from T0 to T1, T1 to T2, and T0 to T2, respectively, in unit of Procrustes distance. The average magnitude of change per-landmark was 1.32 mm, 0.21 mm, and 1.34 mm, respectively. Changes in mean facial form were not statistically significant (*p* = 0.1143). No changes in variance of facial shape were observed across treatment stages (*p* > 0.05).

**Conclusion:**

Rate of facial changes was twice as fast during the first three months as that during fourth to sixth month. Buccal and temporal region became invaginated while labial region became protruded with treatment.

## Background

The importance of a harmonious and aesthetic facial appearance among women is deeply rooted evolutionarily and societally. The number of adult females seeking orthodontic treatment has been on a rise [1]. Improving self-image accounted for more than 70% of adult orthodontic patients’ reasons for seeking orthodontic treatment [2, 3].

Studies on the effect of tooth extraction on facial changes during orthodontic treatment have been inconsistent. Some researchers suggest that face of patients who received tooth extraction became flattened during treatment while it became fuller among non-extraction patients [4, 5]. Bowman and Johnston, on the other hand, suggest that the effect of tooth extraction on facial profile is dependent on initial soft tissue protrusion [6]. It should be noted that these studies were performed based on facial profile of 2D images. Therefore, only facial changes in the midsagittal plane could be evaluated [7]. Taking advantage of 3D facial imaging technology, Moss et al*.* observed complex patterns of facial changes with treatment among adolescent orthodontic patients[8]. In awareness of the complexity of facial structures, Kab et al. divided 3D facial surface into multiple regions for separate analysis [9].

Previous studies of adult facial changes during orthodontic treatment mostly focused on pre- and post-treatment changes of the labial region based on cephalograms. There is currently a lack of understanding of the dynamics of facial changes that take place during the treatment process. In addition, facial changes other than the labial region remain elusive Qin et al. [10] suggested that orthodontic treatment among adults is likely to result in “bracket face” within 1–2 months after treatment, characterized by invaginated cheeks and more prominent zygomatic region which collectively renders a greater-than-actual-age facial appearance. However, without a thorough understanding of facial changes associated with orthodontic treatment, the notion of “bracket face” can hardly be universally accepted.

Conventional facial morphometrics have heavily relied on traditional linear, angular, and proportion measurements [11]. While these measurements are straightforward to understand, each measurement provides only very limited morphometric information. Increasing the number of facial measurements, unfortunately, would make interpretation of key facial changes challenging [12, 13]. Geometric morphometrics (GM) is a revolutionary quantitative morphometric approach based on rigorous statistical theory of shape [14]. Instead of analyzing each facial measurement individually, GM retains the shape and form (shape + size) information encoded by all landmarks during analysis [15]. This facilitates powerful multivariate statistical analysis of facial shape/form and allows for direct visualization of facial shape/form differences.

The use of GM in soft tissue facial analysis is emerging in recent years. Wen et al. [16] investigated facial shape development among Hong Kong adolescents aged 12 to 18 years based on frontal and lateral facial photographs. In the field of orthodontics, Kouli et al. [7] applied GM to investigate changes in facial profile before and after orthodontic treatment. Most current studies are based on 2D images, which are inherently limited in providing a comprehensive understanding of facial changes in 3D. Abedini et al. [17] examined 3D facial changes following micro-implant-supported maxillary skeletal expansion based on facial stereophotogrammetry. However, this study has several methodological limitations. First, the authors’ approach to create left and right expanded group cannot adequately extract the symmetric component of facial shape. As is common in GM, the average of each original configuration and its relabeled reflection (mirrored and labels of bilateral landmarks switched to ensure original left- and right-side landmarks were respectively averaged with reflected right- and left-side landmarks) gives the symmetric component of the configuration [18]. Second, statistical evaluation of treatment-related facial changes was performed without regard to the repeated-measures design of the study, which reduced its power to identify facial regions with statistically significant changes. Third, the exact number of facial landmarks used for analysis was not reported. GM based on spatially dense facial quasi-landmarks (landmarks without distinct anatomical definitions) and the use of appropriate multivariate statistics taking repeated-measures design into consideration are clearly warranted to gain in-depth, high-resolution understanding of natural and treatment-related facial changes.

The present study aimed to apply GM and multivariate statistics to evaluate changes of facial shape and form from baseline through 3 months to 6 months of orthodontic treatment among adult female patients and provide a proof to help orthodontists to realize what “bracket face” actually changed.

## Methods

### Study sample

This study was approved by the Research Ethics Board of the Stomatological Hospital of Chongqing Medical University (No. 2020–013). All patients gave informed consent prior to participation. Patients for this study were recruited from consecutive adult patients visiting the Department of Orthodontics, Stomatological Hospital of Chongqing Medical University, Chongqing, China. A total of 43 females aged 18–26 years were recruited for this study. The mean age of the patients were 21.5 years. Of the 43 patients, 22 received extraction of four first or second premolars prior to the start of orthodontic treatment while 21 patients received orthodontic treatment without tooth extraction.

Patients eligible for this study should satisfy all of the following criteria: adult females of Chinese ethnicity, ANB angle between 0 and 4 degrees, mild to moderate malocclusion, and Body Mass Index within the range of 18.5 to 25 $kg/{m}^{2}$, which represented individuals of normal weight [19]. Patients with obvious facial asymmetry, craniofacial anomalies, previous history of orthodontic treatment, defective dentitions, and significant weight change during treatment were excluded.

All patients were treated with the same fixed appliances (0.022 × 0.028-inch bracket slot). Mandibular bracket was bonded one month after treatment began. The nickel-titanium archwire were changed once a month and the order of the nickel-titanium archwire sequence was 0.012 inch, 0.014 inch, 0.016 inch, 0.016 $\times$ 0.022 inch, and 0.018 $\times$ 0.025 inch.

### Facial surface imaging

Digital facial stereophotogrammetry (Morpheus 3D, Korea) was used to capture 3D facial surfaces for each individual. Patients were imaged following standard facial image acquisition protocol [20]. Patients were asked to gently close mouth, maintain neutral facial expression, and assume natural head position during imaging. Images were taken at three treatment stages: baseline (T0), three months after treatment initiation (T1), and six months after treatment initiation (T2).

### Spatially dense facial quasi-landmarking

**Fig. 1:**
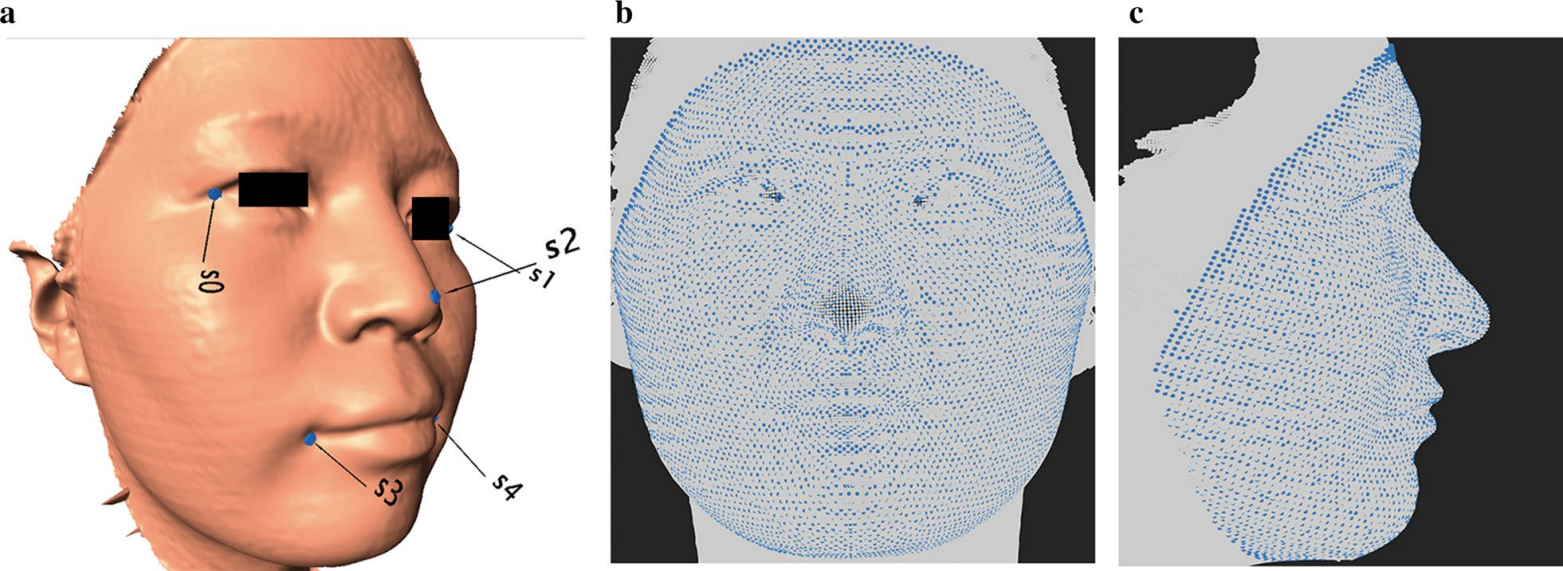
Demonstration of facial mapping. **a** Digitization of five anchoring landmarks. Spatially dense facial landmarks are mapped onto target face as shown in **b** frontal and **c** lateral view

3D facial images obtained from the Morpheus 3D systems was stored in.M3D format, which was converted to the OBJ format by the company. Facial images in OBJ format was further converted to ASCII PLY format. Each facial image in PLY format was imported into the IDAV Landmark Editor v.3.0.0.6 to digitize five anchoring points (right exocanthus, left exocanthus, pronasale, right cheilion, left cheilion) in a fixed order (Fig. 1a) [21].

Facial images in OBJ format, together with coordinates of the five anchoring points, were imported into the MeshMonk toolbox of MATLAB (R2018b) for spatially dense facial quasi-landmarking [22]. Based on the five anchoring points, an anthropometric mask was mapped to each facial image through rigid and non-rigid registration algorithms (Fig. 1b, c). This resulted in 7160 3D facial quasi-landmarks that capture facial region of interest while removing irrelevant structures such as hair, ears, and any dissociated polygons [23].

### Generalized procrustes analysis

Human face is internally symmetric around the midsagittal plane [24]. Each quasi-landmark on the right side had a homologous quasi-landmark on the left side. We reflected and relabeled the quasi-landmark configuration of each patient’s face. All configurations and their relabeled reflections were superimposed through Generalized Procrustes Analysis (GPA). This removed among-configuration variation in size, location, and orientation [25] and resulted in Procrustes shape coordinates that characterized facial shape [26].

Although orthodontic treatment may impact left and right side of the face differentially, this is not the focus of the present study. Therefore, all GM facial analysis in this study was performed based on the symmetric component of facial shape.

### Changes in mean facial shape across treatment stages

Statistical significance of changes of facial shape from T0 through T1 to T2 was evaluated using permutational multivariate analysis of variance (MANOVA) of distance matrices comprising pairwise Procrustes distance (PD) between configurations [27]. Procrustes shape coordinates were used as response variable and treatment stage, treatment modality (tooth extraction vs non-extraction), and their interactions were used as explanatory variables. Unlike traditional MANOVA in which groups under comparison were independent, facial shape at T0, T1, and T2 were correlated because they represented repeated measurements on the same group of patients. Therefore, we constrained permutation to be within each participant, as recommended by Anderson and Braak (2003), to preclude the confounding effect of among-patient variation on evaluations of facial shape changes during treatment [14]. Empirical p values were calculated as the probability that the permuted pseudo F-statistic was larger or equal to the observed F-statistic [27], based on 10,000 permutations. In addition, coefficient of partial determination *(*$partial {R}^{2}$*)* was calculated for each explanatory variable following the formula by Kleinbaum et al. [28].

To gain an understanding of facial regions where shape changed significantly with treatment, we determined statistical significance and relative magnitude of positional changes of each facial quasi-landmark. The analyses were performed based on the same model used above while replacing Procrustes shape coordinates for the entire face with 3D coordinates for each facial quasi-landmark. Similar approach has been adopted by Zaidi et al. [29] and Claes et al*.* [30].

To ascertain the specific treatment stages between which facial shape changed significantly, post-hoc pairwise comparisons were performed. The magnitude of shape change was quantified by PD between mean facial shape of the two treatment stages under comparison. The permutational MANOVA was performed using the Adonis function of the vegan package version 2.5–6 in R version 4.0.0 [31, 32]. 3D Facial heatmaps were used for visualization of study findings.

### Changes in variance of facial shape across treatment stages

Variance of the facial shape was estimated as Procrustes variance [33]. The morphol.disparity function in geomorph package version 3.2.1 in R was used to quantify pairwise differences in variance of facial shape among all treatment stage-by-modality groups [30, 34].

In additional to the above analyses of facial shape, facial form was obtained by multiplying Procrustes shape coordinates of each facial image with its corresponding centroid size. Statistical significance of changes in mean and variance of facial form was performed following the same procedures described above. The level of statistical significance was set at 0.05 for all analyses. Quasi-landmark repeatability error of MeshMonk has been reported to be as low as 0.002 in unit of Procrustes distance, which is robust to manual landmark digitization errors associated with digitization of the five anchoring points [35].

## Result

### Changes in mean facial shape across treatment stages

**Fig. 2:**
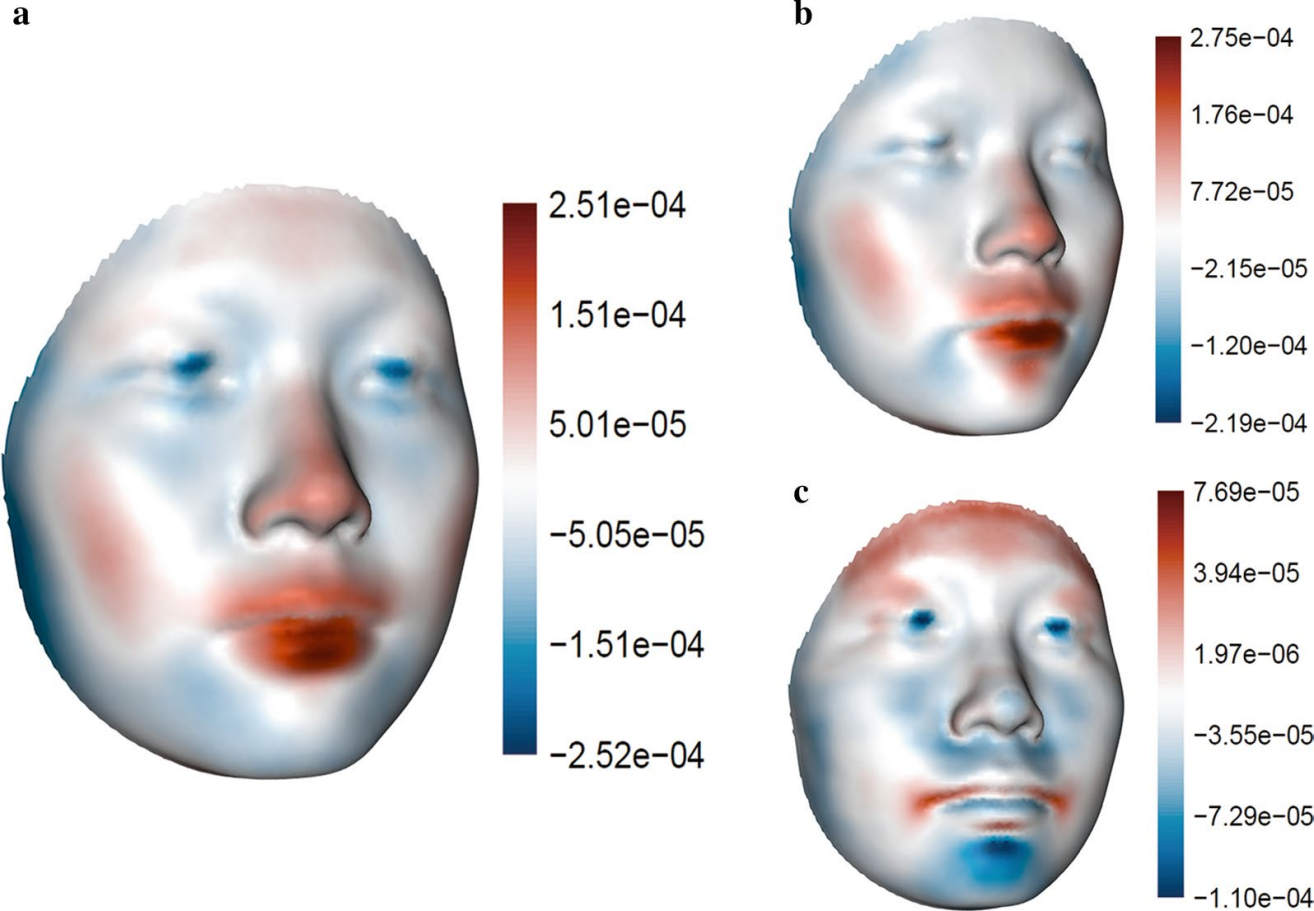
Changes of facial shape during the first six months of orthodontic treatment. Changes of facial shape are illustrated. **a** T0 to T2; **b** T0 to T1; **c** T1 to T2

Permutational MANOVA suggested that changes in mean facial shape were statistically significant across treatment stages (*p* = 0.0022) (Table 1). Pairwise comparisons suggested that facial shape changed significantly from T0 to T2 (*p* = 0.0072) (Table 2). Figure 2a revealed invaginated buccal and temporal region in contrast to protruding labial region with treatment. Pairwise comparison also revealed significant facial changes from T0 to T1 (*p* = 0.0045) (Table 2). Facial changes from T0 to T1 (Fig. 2b) were indistinguishable from changes during T0 to T2 (Fig. 2a). Changes of facial shape from T1 to T2 were characterized by widespread retraction of the mid- and lower-facial third with concomitant perioral protrusion. However, these changes were not statistically significant (*p* = 0.5731) (Table 2). The magnitude of changes in facial shape was 0.009 from T0 to T1, 0.004 from T1 to T2, and 0.010 from T0 to T2 in units of Procrustes distance.

**Table 1:** Main effect of treatment stage, tooth extraction group, and their interaction from permutational MANOVA

	Facial shape		Facial form	
	Partial R^2^	*p* Value	Partial R^2^	*p* Value
Treatment stage	0.00929	0.0022**	0.01551	0.1141
Extraction group	0.06100	0.0051**	0.11565	0.1176
Treatment stage $\times$				
Extraction Group	0.00221	0.3859	0.00294	0.3319

**p < 0.01

Partial R^2^ = coefficient of partial determination

**Table 2:** Pairwise differences in mean shape and form among treatment stages

	Facial shape		Facial form	
	Partial R^2^	*p* Value	Partial R^2^	*p* Value
T0–T1	0.011	0.0045**	0.02113	0.0776
T1–T2	0.0026	0.5731	0.00142	0.6782
T0–T2	0.0151	0.0072**	0.02223	0.0632

**p < 0.01

Partial R^2^ = coefficient of partial determination

**Fig. 3:**
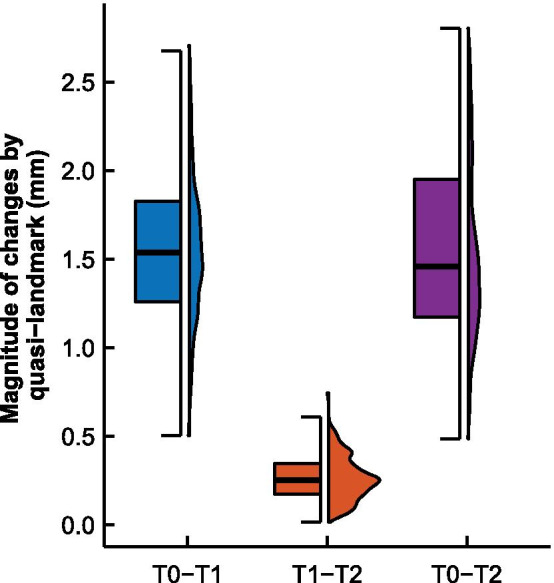
Box plot of distribution of magnitude of changes of quasi-landmarks by treatment stage. **a** T0 to T2; **b** T0 to T1; **c** T1 to T2. Horizontal bar inside box plot indicates the median value. Distribution of magnitude of changes were plotted in histogram next to the box plot

**Fig. 4:**
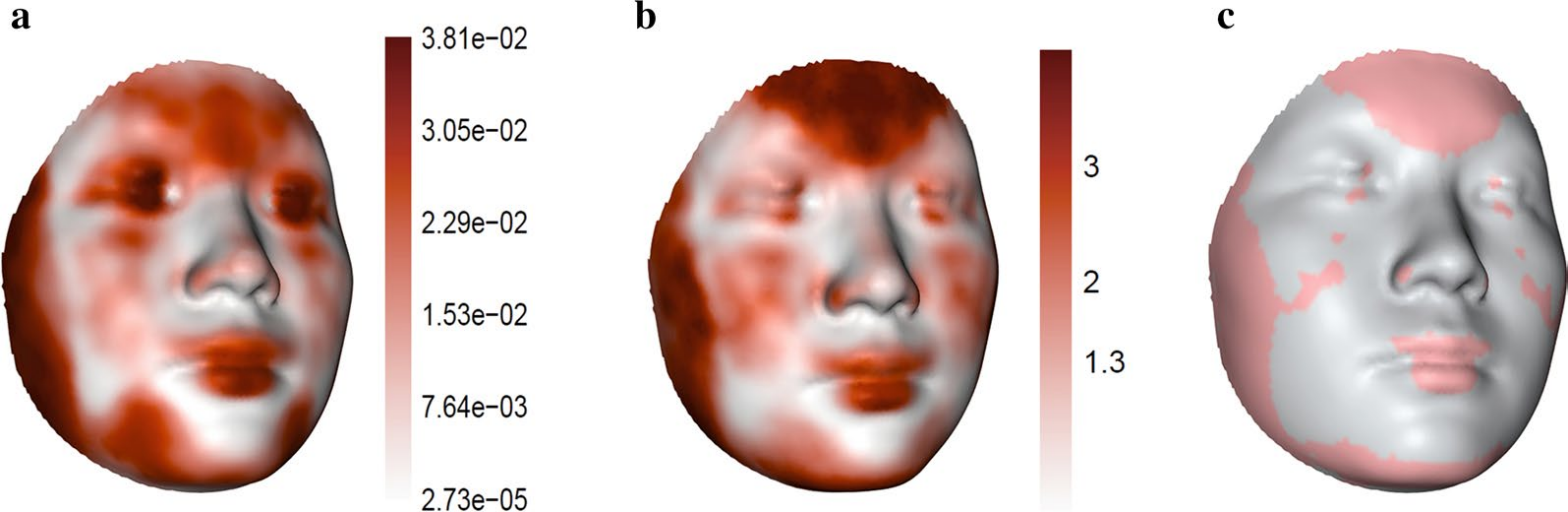
Overall changes of facial shape within the first six months. Heatmap illustrating (**a**) ${R}^{2}$ and (**b**) negative log base 10 of the p value. In **c**, facial regions with significant changes were marked in pink

Distribution of magnitude of changes of facial landmarks was illustrated in Fig. 3. The magnitude of changes was 1.32 mm (SD: 0.38 mm), 0.21 mm (SD: 0.11 mm), and 1.34 mm (SD: 0.45 mm) from T0 to T1, T1 to T2, and T0 to T2, respectively. Quasi-landmarks whose positional changes explained the greatest amount of across-treatment stage facial shape changes were concentrated around buccal, temporal, and labial region. Per-landmark analysis revealed similar patterns of facial changes (Fig. 4) compared to changes suggested by analysis of the entire set of Procrustes shape coordinates (Fig. 2).

### Changes in variance of facial shape across treatment stages

Changes in variance of facial shape was not statistically significant across treatment stages regardless of whether patients received tooth extraction prior to the start of orthodontic treatment (Table 3). Likewise, no changes were observed for variance of facial form across treatment stages in either treatment group (Table 3).

**Table 3:** Pairwise changes in variance of facial shape and form in extraction and non-extraction group

	Extraction group		Non-extraction group	
	ΔVar	*p* Values	ΔVar	*p* Values
*Shape variance*				
T0–T1	0.000321328	0.3614	0.000153771	0.7988
T1–T2	0.000203248	0.6923	0.000014883	0.9653
T0–T2	0.000483553	0.1624	0.000176456	0.8454
*Form variance*				
T0–T1	196,258.7	0.3245	3065.043	0.9189
T1–T2	156.8838	0.9698	394.0572	0.9933
T0–T2	196,473.6	0.3365	2595.986	0.9346

ΔVar: difference in Procrustes variance of facial shape/form between the two time periods under comparison

## Discussion

To our knowledge, this is the first study that investigated longitudinal changes in facial shape/form among adult female orthodontic patients using GM approach based on spatially dense facial quasi-landmarks. Our findings suggest buccal and temporal region invaginated and labial region protruded, in terms of facial shape, as a result of orthodontic treatment. These changes were concentrated within the first three months of treatment. No changes in facial form were observed. Variance of facial shape and form were found constant during the first six months of treatment.

Wirthlin et al. [36] reported an average yearly facial change of 0.15 mm among females from age 22 to 33 years. The average quasi-landmark change of 1.34 mm we observed within the first six months of treatment is a magnitude larger than the amount of natural changes reported. Similarly, Takoma et al. [37] found no significant changes in female facial morphology after age 18 years. Qin et al*.* [10] likewise indicated no significant facial changes among females from 20 to 30 years of age. Therefore, the facial changes we observed is unlikely to be an artefact of natural changes of facial shape that take place during the investigation period.

Both the entire set of facial Procrustes coordinates and per-landmark analysis converged in revealing invaginated buccal and temporal region and protruded labial region. The changes in buccal and temporal regions were likely associated with changes in occlusion and masticatory muscle during orthodontic treatment. As soon as nickel-titanium archwire is ligated, patients report strong pain and tend to avoid chewing [38]. In addition, unstable occlusion during orthodontic treatment further decrease masticatory performance and impair chewing efficiency during initial stages of treatment [39, 40]. Such changes decreased masticatory muscle compliance and leads to gradual muscular atrophy and degradation [41], which consequently likely leads to the buccal and temporal invagination observed in this study.

With regard to the labial region, we observed protruded lips with orthodontic treatment. The amount of protrusion was larger in the lower lips compared to the upper lips. Our findings were inconsistent with the labial retraction identified in a meta-analysis of changes of facial profile during orthodontic treatment [42]. This meta-analysis investigated facial profile change before treatment and after completion of orthodontic treatment. However, our analysis focused on facial changes within the first six months. Space closure after alignment, which usually takes place after around six months of treatment, is likely to result in labial retraction that outweighs the amount of labial protrusion observed during the first six months. Debonding of brackets has been shown to result in labial retraction of 0.23 mm along the direction of midsagittal plane in the lower lip, which is significantly larger than the 0.04 mm change of the upper lip [43]. It is therefore likely that the greater amount of shape change in lower lip during the first six months was due to the greater sensitivity of the lower lip towards bracket attachment. It should also be noted that patients in this study had varying degrees of anterior crowding. The initial proclination during the alignment stage may be another explanation of the observed labial protrusion. However, the relative contribution of bracket attachment and initial proclination to labial protrusion warrants further investigation. This could be achieved by standardization of the degree of anterior crowding during patient recruitment.

The magnitude of facial changes from T0 to T2 (0.010 in unit of Procrustes distance) was smaller than the sum of the magnitude of facial changes from T0 to T1 (0.009) and from T1 to T2 (0.004). The rate of changes of facial shape from T0 to T1 was therefore more than twice the rate of shape change from T1 to T2. Likewise, the average magnitude of per-landmark change from T0 to T2 (1.34 mm) was smaller than the sum of average per-landmark change from T0 to T1 (1.32 mm) and from T1 to T2 (0.21 mm). Analysis of both entire set of Procrustes coordinates and per-landmark analysis therefore converge in suggesting that facial changes were concentrated within the first three months and the patterns of changes of facial shape differed between the first three months and the second three months. The relatively smaller magnitude of changes in dentition may explain decreased rate of shape change from fourth to sixth month. It is quite possible that most of the crowding and irregularities have been corrected in the first three months and chewing efficiency gradually recovered from fourth to sixth month. This may explain why more changes were observed between T0-T1 than T1-T2. Different patterns of changes of facial shape from first to third month and from fourth to sixth month suggested that factors driving changes of facial shape differed during the two periods.

Few studies have investigated variance of facial shape. Among Hong Kong adolescents, variance of frontal facial shape decreased significantly among females but not among males from 12 to 18 years [16]. For lateral facial images, significant reduction in variance of facial shape was observed among both genders. Our study represents the first time orthodontic treatment-related changes in variance of facial shape/form has been investigated. We observed that variance of facial shape remained relatively stable throughout alignment stage of adult orthodontic treatment. Our findings were therefore partially inconsistent with the notion of “bracket face” [10], which should lead to reduce variance of facial shape/form with ongoing orthodontic treatment. However, it remains unclear as to how variance of facial shape would change during space closure and detailing stage of orthodontic treatment.

Tooth extraction was found to have no impact on changes in mean and variance of facial shape/form. Controversies exist as to whether extraction has an impact on changes of facial shape during orthodontic treatment [7, 44]. Well-controlled studies based on adequate sample size and advanced morphometric methods are warranted to provide further evidence in this respect.

Several limitations of the study bear noting. First, there are facial regions beyond those covered by the anthropometric mask that are of theoretical interest. More detailed changes in temporal and buccal region would be investigated if the anthropometric mask could be extended further bilaterally. However, facial regions covered by the anthropometric mask could be reconstructed with high level of fidelity. Our findings therefore provided unprecedented resolution in accurately describing facial shape changes associated with orthodontic changes among adult females. Second, our analysis focused on facial changes during the orthodontic treatment stage of tooth alignment. Longer periods of follow-ups are necessary to gain a complete understanding of facial changes throughout the entire orthodontic treatment. Third, our study sample is comprised of only Chinese. Facial changes and the expectations for orthodontic treatment may differ across ethnic populations [45, 46]. It is therefore meaningful to expand the current methodology to different local populations to generate findings that directly impact treatment of the local population.

Our findings are of clinical significance. Although the present study failed to substantiate the notion of “bracket face”, buccal and temporal invagination and labial protrusion were identified as common changes among adult female orthodontic patients. These changes were concentrated in the first 3 months of treatment and seemed robust to treatment modality (tooth extraction vs non-extraction). Clinical encounter of these facial changes should not be mistaken as unexpected or treatment failure. Orthodontists are advised to be aware of these changes so as to avoid unnecessary adjustment of treatment plan. Furthermore, being cognizant of these changes will allow orthodontists to better communicate with patients so that patients will have a more realistic expectation of the process and outcome of orthodontic treatment.

## Conclusion

Changes of facial shape during the first three months were twice as fast as changes from fourth to sixth month among adult females receiving orthodontic treatment. These changes were characterized by invaginated temporal and buccal regions and protruded labial region. Awareness of these facial changes will help avoid unnecessary adjustment of orthodontic treatment plan and promote communication between orthodontists and patients by helping patients developing a more realistic expectation towards orthodontic treatment.

## Data Availability

The data sets generated and analyzed during the current study are not publicly available due original facial images and coordinates for vertices extracted from MeshMonk software can be used to identify study participants. But are available from the corresponding author on reasonable request.
